# Activity of Antioxidants from *Crocus sativus* L. Petals: Potential Preventive Effects towards Cardiovascular System

**DOI:** 10.3390/antiox9111102

**Published:** 2020-11-09

**Authors:** Keti Zeka, Pasquale Marrazzo, Matteo Micucci, Ketan C. Ruparelia, Randolph R. J. Arroo, Guido Macchiarelli, Stefania Annarita Nottola, Maria Adelaide Continenza, Alberto Chiarini, Cristina Angeloni, Silvana Hrelia, Roberta Budriesi

**Affiliations:** 1 Department of Haematology, Cambridge Biomedical Campus, University of Cambridge, Long Road, Cambridge CB2 0PT, UK; keti.zeka@hotmail.it; 2 Department for Life Quality Studies, Alma Mater Studiorum-University of Bologna, Corso d’Augusto 237, 47921 Rimini, Italy; pasquale.marrazzo2@unibo.it (P.M.); silvana.hrelia@unibo.it (S.H.); 3 Nutraceutical Lab, Department of Pharmacy and Biotechnology, Alma Mater Studiorum, University of Bologna, Via Belmeloro 6, 40126 Bologna, Italy; alberto.chiarini@unibo.it (A.C.); roberta.budriesi@unibo.it (R.B.); 4 Leicester School of Pharmacy, Faculty of Health and Life Sciences, De Montfort University, The Gateway, Leicester LE1 9BH, UK; kruparel@dmu.ac.uk (K.C.R.); rrjarroo@dmu.ac.uk (R.R.J.A.); 5 Department of Life, Health and Environmental Sciences, University of L’Aquila, Via Vetoio, Coppito 2, 67100 L’Aquila, Italy; guido.macchiarelli@univaq.it (G.M.); mariaadelaide.continenza@univaq.it (M.A.C.); 6 Department of Anatomy, Histology, Forensic Medicine and Orthopaedics, La Sapienza University of Rome, 00161 Rome, Italy; Stefania.Nottola@uniroma1.it; 7 School of Pharmacy, University of Camerino, Via Madonna delle Carceri 9, 62032 Camerino (MC), Italy; cristina.angeloni@unicam.it

**Keywords:** cardiovascular prevention, nutraceuticals, crocin, *Crocus sativus* L. petals, kaempferol, by-products

## Abstract

The petals of the saffron crocus (*Crocus sativus* L.) are considered a waste material in saffron production, but may be a sustainable source of natural biologically active substances of nutraceutical interest. The aim of this work was to study the cardiovascular effects of kaempferol and crocin extracted from saffron petals. The antiarrhythmic, inotropic, and chronotropic effects of saffron petal extract (SPE), kaempferol, and crocin were evaluated through in vitro biological assays. The antioxidant activity of kaempferol and crocin was investigated through the 2′,7′-dichlorodihydrofluorescein diacetate (DCFH-DA) assay using rat cardiomyoblast cell line H9c2. The MTT assay was applied to assess the effects of kaempferol and crocin on cell viability. SPE showed weak negative inotropic and chronotropic intrinsic activities but a significant intrinsic activity on smooth muscle with a potency on the ileum greater than on the aorta: EC_50_ = 0.66 mg/mL versus EC_50_ = 1.45 mg/mL. Kaempferol and crocin showed a selective negative inotropic activity. In addition, kaempferol decreased the contraction induced by KCl (80 mM) in guinea pig aortic and ileal strips, while crocin had no effect. Furthermore, following oxidative stress, both crocin and kaempferol decreased intracellular ROS formation and increased cell viability in a concentration-dependent manner. The results indicate that SPE, a by-product of saffron cultivation, may represent a good source of phytochemicals with a potential application in the prevention of cardiovascular diseases.

## 1. Introduction

For centuries, plants have represented the main tools for the treatment of human diseases. In the modern era, phytochemistry and pharmaceutical approaches were applied to investigate the mechanisms of action and the chemical composition of plants used in folk medicine [1,2]. The pharmaceutical value of plant extracts may be due to the synergy of the phytochemicals present in them, even if, rarely, the affection of specific functions may be ascribed to isolated molecules. Phytochemicals are non-nutrients compounds normally found in plants used as food or as components of food supplements, able to exert several biological effects which potentially result in the modulation of several parameters related to human health [3].

Cardiovascular diseases (CVDs) are considered a major cause of mortality worldwide. According to the World Health Organization (WHO), 17.9 million people were estimated to die from CVDs in 2017, representing 31% of all global deaths [4]. Of this mortality, an estimated 7.4 million were due to coronary heart disease and 6.7 million to stroke. In the age of genomics, nanotechnology, and proteomics, CVDs continue to be a major challenge and development of a viable evidence-based way to prevent the onset of CVDs continues to attract the attention of researchers.

Several compounds have been shown to exert biological actions that may result in a decrease of cardiovascular diseases [5,6]. For example, flavonoids can improve cardiovascular health through several mechanisms including the antioxidant effect, the hypocholesterolemic and antidiabetic activities, the antiatherosclerotic and vasorelaxant properties [7,8]. Similarly, carotenoids such as crocin, lutein, zeaxanthin exert biological actions that may determine a plethora of cardiovascular benefits [9,10].

Some spices and vegetal extracts exert antioxidant [9] and hypocholesterolemic effects [11,12]. Saffron (*Crocus sativus* L.) is one of the most popular spices [13]. It is commonly used in regions of Europe such as Italy, Spain, and Greece, and in Asian regions of the world, such as Iran and India [14]. Briefly, saffron is a spice derived from the flower of *Crocus sativus* L (Iridaceae). Each flower has three stigmas, which are dried and used in cooking as a seasoning and as a coloring agent. They contain a carotenoid dye, crocin, which gives food a rich golden-yellow hue [7,15,16]. Saffron consumption is shown to correlate with higher High Density Lipoproteins (HDL) and lower triglycerides levels [17]. Moreover, administration of 15 mg of saffron capsules to diabetic patients for a 3 month period resulted in a significant decrease of fasting plasma glucose, cholesterol, Low Density Lipoproteins (LDL-c), and LDL/HDL ratio in comparison to the placebo [18].

**Figure 1:**
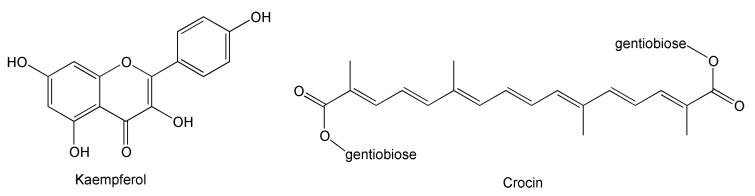
Chemical structures of kaempferol and crocin.

To date, well over 6500 publications listed in the PubMed Central database demonstrate the potential uses of saffron extracts as adjuvant in anti-cancer, anti-diabetic, anti-depressant, and anti-inflammatory treatment. Our group has shown the presence of substantial amounts of kaempferol and crocin (Figure 1) in saffron petals [15,16] which make up the bulk of the flowers, but are currently considered a waste material.

Kaempferol is one of the main flavonols of the saffron spice and has protective properties against lipid oxidation, which contributes to the onset and progression of atherosclerosis and cardiovascular diseases [7,8]. Originally derived from the carotenoid crocetin, crocins have a common structural feature of digentiobiose *O*-glycosyls [19]. Crocin contributes to the bitter taste of saffron and to its characteristic yellowish color [20,21].

In this work, we aim to investigate the cardiovascular effects of saffron petal extract (SPE) and its main components kaempferol, crocin and the effects these compounds have on the oxidative state of rat cardiomyoblast cell line H9C2.

## 2. Materials and Methods

### 2.1. Chemistry

2′,7′-dichlorodihydrofluorescein diacetate (DCFH-DA), 3-(4,5-dimethylthiazol-2-yl)-2,5-diphenyl-tetrazolium bromide (MTT), Dulbecco’s modified Eagle’s medium (DMEM), fetal bovine serum (FBS), penicillin/streptomycin, l-glutamine (Sigma Aldrich—Merck Darmstadt, Germany).

#### 2.1.1. Plant Material

Saffron petals were collected on October 2018 from the plains of Navelli, the center of saffron production in the Abruzzo region, Italy. The plants are a designated DOP (“Protected Designation of Origin”). Petals were obtained after the stigmas had been manually removed from the saffron flowers. Fresh petals were oven dried (o/n at 65 °C) and stored in batches of 200 g packed in vacuum sealed plastic bags, in the dark, at room temperature.

#### 2.1.2. Extraction and Purification

Oven-dried petals (200 g) were coarsely ground and left to macerate in methanol (4 × 500 mL) for 48 h at room temperature until complete discoloration of petals. The methanol was removed by evaporation at 30 °C under reduced pressure, and the dry residue stored as total saffron petals extract (SPE), in glass vials at −20 °C. Some SPE was used for further purification of kaempferol and crocin as described before [15].

#### 2.1.3. Chemical Analyses

^1^H-NMR and ^13^C-NMR spectra were recorded on a Bruker 400MHz super-conducting spectrometer at 30 °C. Tetramethylsilane was used as an internal standard for ^1^H-NMR spectra. Infrared spectra (IR) were recorded on a Perkin-Elmer 298 spectrophotometer. Solid samples were prepared as KBr discs and liquids as thin films using NaCl plates.

Mass spectra were recorded on a Micromass Quattro II low resolution triple quadruple mass spectrometer by the EPSRC National Mass Spectrometry Service Centre (Swansea, UK).

Melting points were determined using a Gallenkamp melting point apparatus and are uncorrected.

Thin layer chromatography (TLC) was carried out using Merck aluminum sheet-silica gel 60F254 coated plates which were visualized under UV light and stained with either iodine, 2,4-dinitrophenylhydrazine, or phosphomolybdic acid.

Reagents were used as received from Sigma-Aldrich Chemical Company (Dorset, UK).

#### 2.1.4. Quantification of Crocin and Kaempferol

Extracts of petals were used to quantify crocin and kaempferol by areas comparison under the peak with the authentic reference samples. An Agilent 1100 Series HPLC, equipped with diode array detection was used for the quantification of crocin and kaempferol. The column used was Hichrom ACE Ultracore 5 Super C_18_ (150 × 4.6 mm, ID). Flow rate was 1 mL/min, sample size was 5 μL. Spectra were recorded from 190 to 400 nm as reported previously [15].

### 2.2. Ex Vivo Guinea Pig Assay

Male guinea pig (200–400 g) obtained from Charles River (Calco, Como, Italy) were used. The animals were housed according to the ECC Council Directive regarding the protection of animals used for experimental and other scientific purposes. The work was conducted according to the guidelines set forth by EU Directive 2010/63/EU. The protocol was approved by the Institutional Ethics Committee of the University of Bologna (Protocol PR 21.79.14) and transmitted to the Ministry of Health. The animals were sacrificed by cervical dislocation. The used organs were heart, vascular (aorta) and non-vascular (ileum) smooth muscle. Briefly, the organs were set up rapidly under a suitable resting tension in 15 mL organ bath, containing appropriate physiological salt solution (PSS) consistently warmed and buffered to pH 7.4 by saturation with 95% O_2_–5% CO_2_ gas and used through specific protocols.

#### 2.2.1. Atria

Isolated guinea pig heart preparations were used: spontaneously beating right atria and left atria driven at 1 Hz. The method was previously described [22].

#### 2.2.2. Aortic Strips

The thoracic aorta was removed and placed in Tyrode solution as previously reported [22].

#### 2.2.3. Ileum

The method was previously described [23]. Briefly, the terminal portion of the ileum (2–3 cm near the ileo-cecal junction) was cleaned, and segments 1 cm long of the ileum were set up under 1 g tension at 37 °C in organ baths containing Tyrode solution of the following composition (mM): NaCl 118, KCl 4.75, CaCl_2_ 2.54, MgSO_4_ 1.20, KH_2_PO_4_·2H_2_O 1.19, NaHCO_3_ 25, and 11 glucose equilibrated with 95% O_2_–5% CO_2_ gas at pH 7.4. The intestine was removed above the ileo-cecal junction. Segments of 2 cm length were mounted under a resting tension of 300–400 mg. Strips were secured at one end to a force displacement transducer (FT 0.3, Grass Instruments Corporation) for monitoring changes in isometric contraction.

#### 2.2.4. Statistical Analysis

Data from different isolated tissue preparations were analyzed by the Student’s *t*-test and presented as means ± S.E.M [24] or by one-way ANOVA followed by Dunnett’s test and *p* value less than 0.05 has been considered significant. The potency of SPE and isolated compounds defined as EC_50_ and IC_50_ were calculated from concentration–response curves (Probit analysis using Litchfield and Wilcoxon [24] (Springer, New York, NY, USA) or GraphPad Prism^®^ [25,26] software (GraphPad Prism 5.02 Software, San Diego, CA, USA, www.graphpad.com).

### 2.3. In Vitro Antioxidant Activity Assay

#### 2.3.1. Cell Culture and Treatments

H9c2 rat myoblast cell line was obtained from the European Collection of Cell Cultures (Salisbury, UK). Cells were cultured in Dulbecco’s modified Eagle’s medium (DMEM) 10% (*v/v*) heat-inactivated FBS, penicillin (100 U/mL), streptomycin (100 mg/mL), and l-glutamine (2 mM) and grown at 37 °C in an atmosphere of 5% CO_2_ and 95% air and split 1 to 4 at sub-confluence (80%). Cells were seeded in 96-well cell culture plates at 50,000 cells/mL in 100 μL per well.

Crocin and kaempferol were solubilized in DMSO. All treatments were carried out using the same amount of DMSO (0.1%) that does not influence cell viability (data not shown). The same amount of DMSO was added to control cells. The day after seeding, the cells were treated for 1 or 24 h with 0.1–100 µM crocin or kaempferol.

#### 2.3.2. ROS Detection

For intracellular ROS detection, 2′,7′-dichlorodihydrofluorescein diacetate (DCFH-DA) assay was used, as previously reported [27]. Pre-treated cells were incubated with 10 μM DCFH-DA in DMEM without phenol red for 30 min. After DCFH-DA removal, cells were incubated with 100 μM H_2_O_2_ in PBS for 15 min. Cells were washed with PBS and the fluorescence was measured using 485 nm excitation and 535 nm emission with a microplate spectrofluorometer (VICTOR3 V Multilabel Counter, PerkinElmer, Wellesley, MA, USA). Intracellular ROS were also evaluated at single cell level by flow cytometry. A number of 2 × 105 cells was seeded in 12-well tissue culture plates and left in culture overnight. The cells were treated with crocin or kaempferol (5 μM) for 1 h in DMEM 10% FBS at 37 °C 5% CO_2_, then stained with 10 μM DCFH-DA in DMEM without phenol red and FBS for 30 min, 37 °C 5% CO_2_. The cells were detached with trypsin solution and centrifuged at 300× *g* for 5 min, in 1.5 mL tubes. After removing the supernatant, the cell pellet was resuspended and appropriately diluted to 5 × 10^−5^ cells/mL in PBS. Guava^®^ easyCyte™ 5 HT instrument was used to collect flow cytometry data. FlowJo software was used to analyse the geometric mean fluorescence intensity (MFI). Unstained untreated samples were used as controls.

#### 2.3.3. Cell Viability Assay

For cell viability detection, 3-(4,5-Dimethylthiazol-2-yl)-2,5-diphenyl-tetrazolium bromide (MTT) assay was used, as previously reported [28] pre-treated cells were treated with 100 μM H_2_O_2_ in PBS for 45 min, at 37 °C 5% CO_2_. After aspirating the wells, MTT (Sigma Aldrich—Merck Darmstadt, Germany) was incubated in growth medium and MTT solution was added to the cells at 0,5 mg/mL for 1.5 h 37 °C 5% CO_2_. After removing MTT solution, DMSO was added to dissolve formazan in the cells. The absorbance of formazan was measured spectrophotometrically at 595 nm using VICTOR3 V Multilabel plate-reader (PerkinElmer, Wellesley, MA, USA). Untreated cells were considered as viability control in the assay.

#### 2.3.4. Total Antioxidant Activity (TAA)

Total antioxidant activity was determined in H9c2 cells using the method of Re et al. [29], based on the ability of the antioxidant molecules in the sample to reduce the radical cation of ABTS, and measured as quenching of the absorbance at 740 nm, as previously reported [30]. Briefly, H9c2 cells were washed 3 times with cold phosphate buffer solution (PBS). Cells were subsequently collected in 2 mL of PBS and cells were lysed with two freeze–thaw cycles. H9c2 were centrifuged for 10 min at 5000× *g* at 4 °C, after which the supernatant was collected. Values obtained for each sample were compared to the concentration–response e curve of a standard Trolox solution, calculated as Trolox equivalent antioxidant activity (TEAA)/mg of protein and express as % of control.

#### 2.3.5. Real-Time Polymerase Chain Reaction (PCR) Assay

Total RNA was extracted using RNeasy mini kit (QIAGEN GmbH, Hilden, Germany), following the manufacturer’s protocol. The yield and purity of the RNA were measured using NanoVue spectrophotometer (GE Healthcare, Milano, Italy). One μg of total RNA was reverse-transcribed to cDNA using iScript cDNA synthesis kit (BIO-RAD, Hercules, CA, USA), following the manufacturer’s instructions. The subsequent PCR was performed in a total volume of 10 μL containing 2.5 μL (12.5 ng) of cDNA, 5 μL SsoAdvanced Universal SYBR Green Supermix (BIO-RAD) and 0.5 μL (500 nM) of each primer. The primers used (SIGMA-ALDRICH, Milan, Italy) are reported in Table 1; actin was used as reference gene for H9c2 cells.

## 3. Results

### 3.1. Chemistry

SPE had been chemically characterized [15]. Alongside fibers, fats, proteins, and minerals, Navelli saffron crocus contains significant quantities of secondary metabolites such as kaempferol. In particular, kaempferol glycosides were shown to be abundant in dried petals at 126 mg/g DW, while crocin concentration was 6.4 mg/g DW [15]. Both kaempferol and crocin were isolated to a level exceeding 99% purity, as confirmed by HPLC-DAD and NMR.

### 3.2. Effects of SPE, Crocin and Kaempferol towards Cardiac and Smooth Muscle System

SPE and the isolated compounds, crocin and kaempferol, were tested for their cardiac profile on guinea pig left atrium driven at 1 Hz and on spontaneously beating right atrium to evaluate their inotropic and chronotropic effects, respectively. Data of cardiac activity for extract and compounds are collected in Table 2.

**Figure 2:**
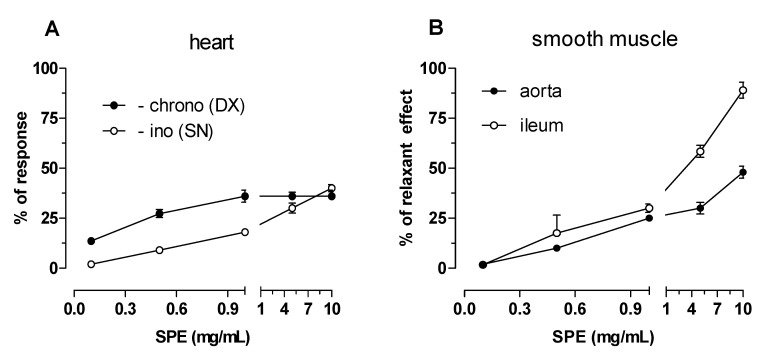
(**A**) Negative inotropic and chronotropic effects on guinea pig left and right atrium respectively. Cumulative concentration–response curves for Saffron Petal Extract (SPE) were obtained using negative effects as percentage of the initial tension (left atria) and initial rate (right atria). (**B**) Spasmolytic effect of SPE on 80 mM K^+^-induced contraction on guinea pig vascular (aorta) and non-vascular (ileum) smooth muscle. Cumulative concentration–response curves for SPE were obtained using relaxant effects as percentage of the initial tension induced by 80 mM K^+^, taken as 100%. Each point is the mean ± SEM (*n* = 5–6). Where error bars are not shown these are covered by the point itself.

As shown in Table 2 and Figure 2A, SPE exerted weak negative inotropic and chronotropic effects. The maximum negative inotropic (36 ± 0.9%) and negative chronotropic (40 ± 1.4%) effects were reached at 1 and 10 mg/mL, respectively.

SPE, crocin, and kaempferol were tested on K^+^-depolarized (40 or 80 mM) guinea pig smooth muscles: aortic strips and ileum longitudinal smooth muscle to assess their vascular- or not vascular-relaxant activity, respectively. Data are included in Table 2.

On vascular smooth muscle, SPE had intrinsic effect less than 50% (48 ± 1.3%) at 10 mg/mL against KCl-induced contraction (Figure 2B). On the contrary, SPE inhibited K^+^ 80 mM-induced contraction on guinea pig non-vascular smooth muscle. The maximum effect (89 ± 1.9%) was reached at 10 mg/mL with a potency of 2.05 mg/mL (c.l. 1.36–3.10).

**Figure 3:**
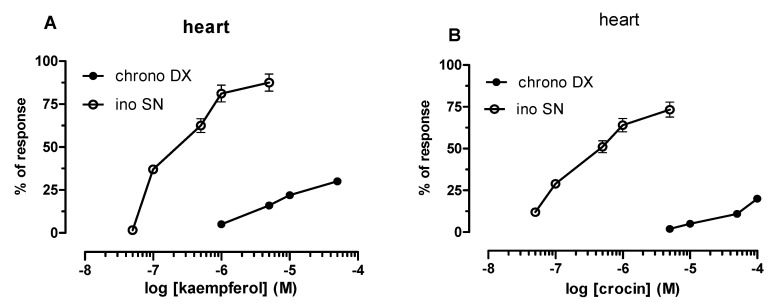
Negative inotropic and chronotropic effects on guinea pig left (ino SN) and right atrium (chrono DX) respectively. Cumulative concentration–response curves for kaempferol (**A**) and crocin (**B**) were obtained using negative effects as percentage of the initial tension (left atria) and initial rate (right atria). Each point is the mean ± SEM of four–six experiments (*n* = 4–6). Where error bars are not shown these are covered by the point itself.

Crocin and kaempferol showed an interesting cardiac profile: a selective negative inotropic activity (Figure 3).

The maximum intrinsic effect is achieved at 5 µM for both compounds (Table 2). They have negative inotropic potency in the 0.1 µM range [EC_50_ = 0.17 µM (c.l. 0.12–0.23); EC_50_ = 0.15 µM (c.l. 0.083–0.28) respectively]. This negative inotropic effect is selective of the left atrium driven at 1 Hz; in fact, it does not occur in the spontaneously beating right atrium where the intrinsic activity is around 30% for both compound (Table 2). The same can be said of the negative chronotropic effect (Table 2).

**Figure 4:**
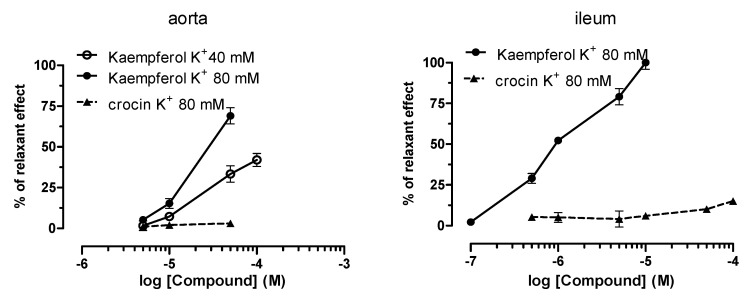
Spasmolytic effect of crocin and kaempferol on high K^+^-induced contraction on guinea pig vascular (aorta) and non-vascular (ileum) smooth muscle. Cumulative concentration–response curves for crocin and kaempferol were obtained using relaxant effects as percentage of the initial tension induced by 40- or 80-mM K^+^, taken as 100%. Each point is the mean ± SEM (*n* = 5–6). Where error bars are not shown these are covered by the point itself.

Crocin and kaempferol were tested on K^+^-depolarized (80 mM) guinea pig smooth muscles: i.e., aortic strips and ileum longitudinal smooth muscle, in an attempt to assess their vascular- or non-vascular-relaxant activity respectively. The data are presented in Table 3 together with those of SPE. Crocin had no effect on vascular or non-vascular smooth muscle (Figure 4).

In contrast, kaempferol inhibited K^+^-induced contraction on guinea pig vascular and non-vascular smooth muscle (Figure 4). In particular, kaempferol showed a similar intrinsic activity, but the maximum effect is reached at concentrations 10 times lower than for the non-vascular smooth muscle [(69 ± 1.9%) at 50 µM, (79 ± 2.4%) at 5 µM, respectively]. Kaempferol has non-vascular relaxant potency seventeen times greater than on vascular smooth muscle [EC_50_ = 20.76 µM (c.l. 12.58–29.37); EC_50_ = 1.16 µM (c.l. 7.74–2.36); respectively]. On aorta K^+^ 40 mM depolarized, kaempferol show an intrinsic activity of less than 50% (42 ± 1.6%) at 50 µM (Figure 4).

The intrinsic activity decreases when the K^+^ concentration is reduced to 40 mM. This finding is in agreement with the assumption that the calcium channels blocking by specific drugs is linked to membrane depolarization [31,32]. It is well-known that classic Ca^2+^ channel blockers such as nifedipine, verapamil, and diltiazem act in this way, though some natural extracts and derivative compounds seem to act in the same way [31,32].

### 3.3. Effects of Crocin and Kaempferol on H9c2 Cellular Viability

**Figure 5:**
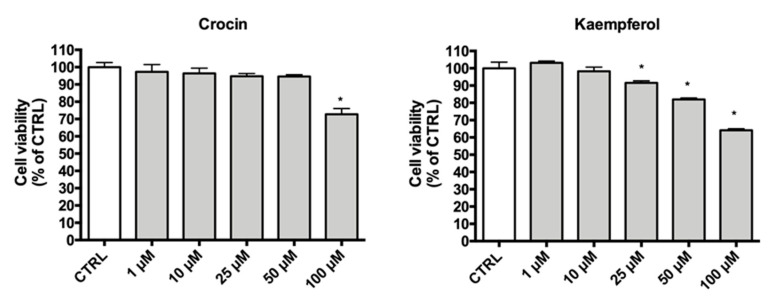
Viability of H9c2 cells treated with crocin and kaempferol. Cells were treated for 24 h with increasing concentrations (1–100 µM) of crocin or kaempferol. Viability was measured by MTT assay, as reported in Materials and Methods. Each bar represents means ± S.E.M. of at least 4 independent experiments. (*n* = 4) Data were analyzed by one-way ANOVA followed by Dunnett’s test. * *p* < 0.05 with respect to CTRL.

H9c2 cells were treated with different concentrations (1–100 µM) of crocin and kaempferol for 24 h. Data reported in Figure 5 show that crocin is cytotoxic only at the highest concentration. Kaempferol showed a higher cytotoxicity, in fact, cell viability was significantly lower in respect to control cells at concentrations equal or greater than 25 µM. For this reason, the next experiments were carried out using concentrations lower than 25 µM.

**Figure 6:**
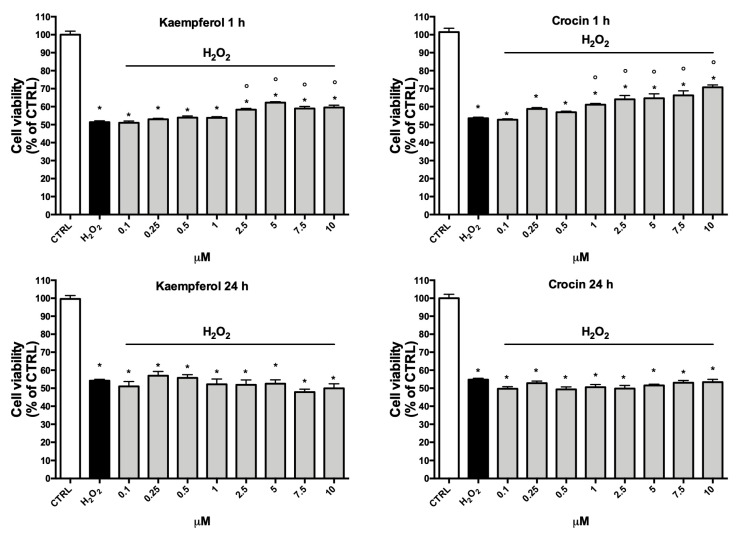
Effect of crocin and kaempferol against H_2_O_2_-induced injury in H9c2 cells. Cells were pre-treated with crocin or kaempferol (0.1–10 μM) for 1 or 24 h and then exposed to 100 µM H_2_O_2_. Cell viability was measured by MTT assay as reported in Materials and Methods. Data are reported as % increase in respect to H_2_O_2_ treated cells. Each bar represents means ± SE of at least four independent experiments (*n* = 4). Data were analyzed by one-way ANOVA followed by Dunnett’s test. * *p* < 0.05 H_2_O_2_.

To investigate the potential protective effect of the two compounds against oxidative stress, cells were treated with increasing concentrations (0.1–10 µM) of crocin or kaempferol for 1 h or 24 h before the induction of oxidative stress (100 µM H_2_O_2_) (Figure 6). This peroxide concentration has been chosen as it reduces cell viability by 50% with respect to control cells. Moreover, similar H_2_O_2_ concentrations had been used by other authors [33] in H9c2 cells. Treatment with 1–10 µM crocin for 1 h was able to counteract peroxide-induced damage, significantly increasing cell viability in respect to H_2_O_2_ treated cells. Kaempferol treatment for 1 h was less effective than crocin and increased cell viability after peroxide-induction only at concentrations of 2.5 µM and higher, moreover the highest % increase triggered by kaempferol was only 9% at 5 µM, whereas 10 µM crocin led to a 16% increase in respect to H_2_O_2_. Interestingly, the long-term treatments (24 h) did not show any protective effect, suggesting that these compounds may act with a direct antioxidant mechanism.

**Figure 7:**
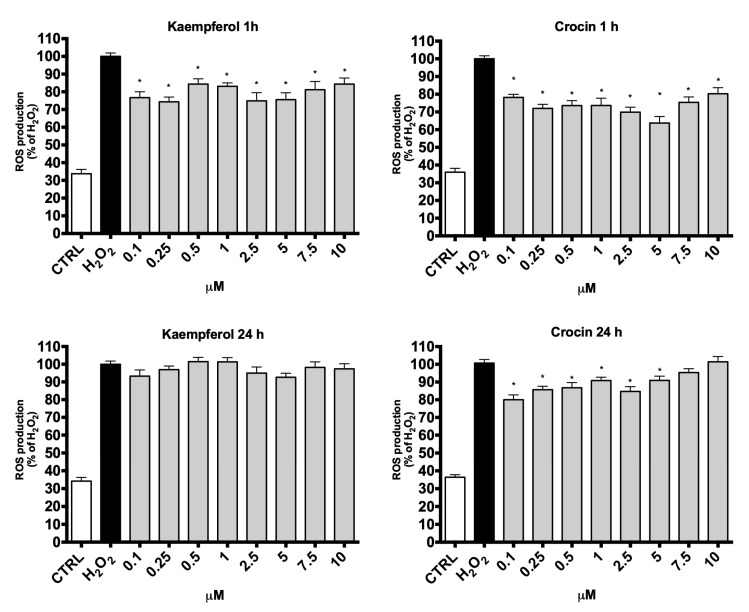
Effect of crocin and kaempferol on Reactive Oxygen Species (ROS) production in H9c2 cells. Cells were pre-treated with crocin or kaempferol (0.1–10 μM) for 1 or 24 h and then exposed to 100 µM H_2_O_2_. Intracellular ROS production was measured with the peroxide-sensitive probe DCFH-DA as reported in Materials and Methods. Data were expressed as percentage with respect to H_2_O_2_-treated cells. Each bar represents means ± SE. of at least four independent experiments (*n* = 4). Data were analyzed by one-way ANOVA followed by Dunnett’s test. * *p* < 0.05 with respect to H_2_O_2_.

To better investigate this aspect, H9c2 cells were treated with 0.1–10 µM crocin and kaempferol for either 1 or 24 h prior to the addition of H_2_O_2_ and the level of intracellular ROS was determined using the peroxide-sensitive fluorescent probe DCFH-DA (Figure 7). Regarding the short-term treatments (1 h), unlike the data on the protection against H_2_O_2_, all tested crocin and kaempferol concentrations led to a significant reduction of endogenous ROS levels in H_2_O_2_ treated cells. This discrepancy may be due to the different sensitivity of the two assays. In agreement with the MTT data, the long-term treatments (24 h) were less effective in reducing ROS levels in respect to 1 h treatments. In particular, all the tested concentrations of kaempferol were not able to reduce ROS levels in respect to peroxide. On the other hand, crocin at 0.1–5 µM significantly reduced ROS levels in respect to H_2_O_2_.

**Figure 8:**
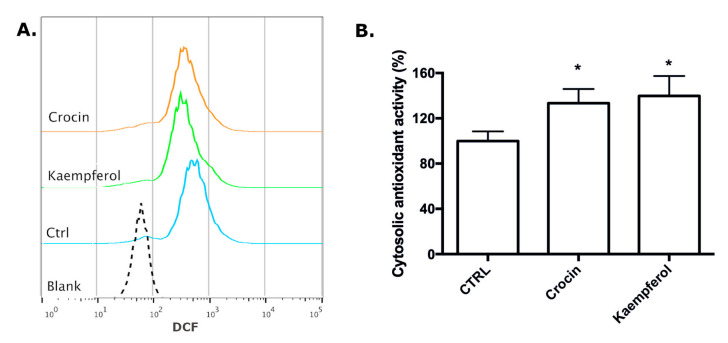
Effect of crocin and kaempferol on ROS production at single cell level and total antioxidant activity of H9c2 cells. H9c2 cells were treated with 5 μM of the crocin or kaempferol for 1 h. (**A**) Intracellular ROS were evaluated at single cell level using DCFH-DA by flow cytometry as reported in Materials and Methods. (**B**) Total antioxidant activity was measured as reported in the Materials and Methods and expressed as percent of control cells. Each bar represents means ± SE of three independent experiments (*n* = 3). Data were analyzed with one-way ANOVA followed by the Bonferroni’s test. * *p* < 0.05 vs. CTRL.

Intracellular ROS were also evaluated at the single cell level in basal condition, i.e., without exposing cells to H_2_O_2_, by flow cytometry (Figure 8A). H9c2 cells were treated with 5 µM crocin and kaempferol for 1 h and stained with DCFH-DA before flow cytometry analysis. In agreement with the previous data, the peaks relating to the cells treated with either crocin or kaempferol had shifted to the left away from the positive control, towards that of the negative control (blak). This is due to the lower intracellular fluorescence of the DCFH-DA dye compared to positive control cells, indicating a lower level of ROS.

To confirm the ability of the two compounds to scavenge ROS, H9c2 cells were treated with 5 µM crocin and kaempferol for 1 h and total antioxidant activity (TAA) was evaluated (Figure 8B). As expected, both crocin and kaempferol were able to significantly increase TAA, in agreement with the data on the ROS production.

**Figure 9:**
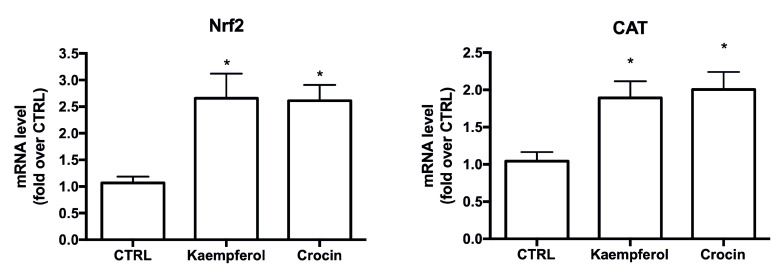
Effect of kaempferol and crocin on Nrf2 and antioxidant enzyme catalase (CAT) expression. Real time-PCR was performed to detect Nrf2 and CAT mRNA levels. Data are expressed as relative abundance compared to untreated cells. Each bar represents mean ± SEM of three independent experiments. Data were analyzed with one-way ANOVA followed by the Bonferroni’s test. * *p* < 0.05 vs. CTRL.

In order to better clarify the mechanisms behind crocin and kaempferol protection, expression of the Nrf2/ARE signaling pathway was investigated. In particular, RT-PCR analysis was carried out to determine if the compounds modulate the expression of Nrf2 and the antioxidant enzyme catalase (CAT). Cells were treated with kaempferol and crocin 5 µM for 1 h before RT-PCR analysis (Figure 9). Of note, both crocin and kaempferol were able to significantly up-regulate both Nrf2 and catalase, suggesting their involvement in the Nrf2/ARE pathway.

## 4. Discussion

Nowadays, the relationship between cardiac dysfunction and initiating factors such as smoking, excessive alcohol consumption, diet, obesity, and stress is well known. In several cases, the effect of one of these factors alone or in combination with others, as atherosclerosis and hypertension, represents a first step in the development of different cardiac dysfunctions such as angina, infarction, arrhythmias, or heart failure [34].

Dietary factors play a key role in the development as well as prevention of certain human diseases, including cardiovascular diseases. Currently, there has been increased global interest in identification of medicinal plants that are pharmacologically effective and have little or no side effects for use in preventive medicine. There is a growing amount of literature concerning the potential benefits of these herbs and spices from a health perspective especially in conferring protection against cardiovascular diseases.

Many phytochemicals have been shown to exert antioxidant effect and to counteract many oxidative-stress-related diseases, like cardiovascular diseases [35,36].

In accordance with principles of circular economy, we focused on agricultural waste products of a crop cultivated in Italy, with the aim to provide a health promoting substance for nutraceutical application.

*Crocus sativus* L. has been used for centuries in folk medicine, for the treatment of several disorders, including those concerning cardiovascular system [13]. Our group has demonstrated lately the high antioxidant potential of kaempferol and crocin [15].

In the current work, we report its ability to affect cardiovascular parameters by influencing inotropy, chronotropy and to reduce KCl induced contraction in vascular and non-vascular smooth muscle and its antioxidant activity in cardiac cells.

An aqueous extract of *Crocus sativus* L. petals reduced blood pressure in normotensive and hypertensive anesthetized rats, though no active pharmaceutical ingredient was identified [37] as this compound was shown to prevent angiotensin II-induced hypertension in anesthetized rats [38]. Our data suggest that crocin does not directly affect smooth muscle contractility, as it has not reduced the contractions induced by KCl in guinea pig vascular (aorta) and non-vascular (ileum) smooth muscle. The hypotensive activity was also observed with a *Crocus sativus* L. hydroalcoholic extract in angiotensin II hypertensive rats, likely due to the inhibition of renin-angiotensin system [39]. The hypotensive activity of saffron extract may also occur through the inhibition of calcium channels, the reduction of calcium release into sarcoplasmic reticulum [40], and through the antagonism towards adrenergic receptors [41]. Although previous reports demonstrated that some saffron extracts and related compounds act on vascular smooth muscle, no data on extracts from saffron petals from Navelli, L’Aquila (Italy) are available.

In our work, SPE exerted a selective spasmolytic effect towards 80 mM K^+^-induced contraction in guinea pig non-vascular (ileum) smooth muscle, suggesting the involvement of calcium channels. This is in agreement with the observation that saffron extract has the ability to reduce calcium influx inhibiting receptor operated Ca^2+^ channels [37].

A similar effect was observed with crocin, while kaempferol had a relaxant effect both on guinea pig vascular (aorta) and non-vascular (ileum) smooth muscle, showing a higher potency towards the latter tissue.

The maintenance of cardiovascular health is the result of multiple molecular events that concern, among others, the state of inflammation and oxidative stress. Therefore, in addition to the study of the parameters described above, we measured the antioxidant effect of the products derived from *Crocus* on the oxidative state of H9c2 cells.

Our data show that crocin and, to a lesser extent, kaempferol protect H9c2 cells against H_2_O_2_-induced damage by reducing intracellular ROS levels and thus counteracting oxidative stress. The antioxidant activity of crocin and kaempferol purified from petals of *Crocus sativus* L. has already been demonstrated in vitro [15]. There, the authors used two different antioxidant tests: DPPH and ABTS assays. Both showed that the compounds have antioxidant activity, kaempferol being more effective than crocin. In contrast, our current data on the in vitro cell-based assay show a higher effect of crocin compared to kaempferol in reducing ROS levels and our hypothesis is that this could be due to a different cellular uptake of the two compounds. Crocin has been demonstrated to penetrate the blood brain barrier and localize inside the brain after i.p. administration in mice [42], suggesting its bioavailability in vivo. On the other hand, kaempferol is poorly absorbed due to its low water solubility [43].

Our data on the antioxidant activity of crocin are in agreement with those of previous experiments [44] where crocin attenuated the effects of LPS-induced expression of inflammatory factors and oxidative stress. In a cell model of ischemia/reperfusion, 10 µM crocin for 24 h increased Nrf2 and HO-1 levels in rat cardiomyocytes [45], suggesting that crocin not only acts with a direct antioxidant mechanism but also with an indirect one through the upregulation of endogenous antioxidant enzymes.

The antioxidant activity of kaempferol has also been investigated against antimycin A-induced oxidative stress in osteoblast-like MC3T3-E1 cells [46]. The authors demonstrated the reduction of ROS level using both DCFH-DA and MitoSOX, a live-cell-permeable and mitochondrial localizing superoxide indicator. In H9c2 cells, 2.5 µM kaempferol for 1 h reduced oxygen-glucose deprivation (OGD)-triggered cell damage through down-regulating miR15b expression via activating PI3K/AKT and Wnt3a/β-catenin pathways [47]. Of note, OGD triggers oxidative stress, so the protection evoked by kaempferol could also be related to its antioxidant activity [48].

Interestingly, our data show that crocin and kaempferol short-term treatments (1h) are more effective than long-term treatments (24 h). In particular, a long-term treatment with kaempferol did not show any protective effect, meanwhile, a long-term treatment with crocin did not show any protective effect in terms of cell viability but was able to slightly reduce intracellular ROS level. This could be due to the molecular mechanisms by which the compounds counteract oxidative stress. In particular, the fact that only the short-term treatment counteracts oxidative stress let us speculate a direct antioxidant mechanism, that could not be present anymore after a long term-treatment. In fact, after 24 h, the compounds might be extensively metabolized. To clarify this point and understand if the compounds act through a direct scavenging activity of ROS or by enhancing the endogenous antioxidant system, we investigated the level of the transcription factor Nrf2 and the antioxidant enzyme catalase after 1 h treatment. Both the compounds were able to up-regulate Nrf2 and catalase, suggesting that they also act with an indirect mechanism, enhancing the antioxidant defense system. Further studies will be necessary to understand the potential additive or synergic effect of the compounds used in combination. Kaempferol and crocin exerted significant antioxidant activity in cardiac cells. In agreement, the antioxidant activity of those compounds was previously observed by the ABTS and DPPH tests [15,36].

## 5. Conclusions

*Crocus sativus* L. petals are a source of bioactive compounds such as kaempferol and crocin. Both SPE and isolated compounds may find use as nutraceuticals to prevent or reduce the effects of non-communicable diseases, if applied in a clinical space “beyond diet, before drug”. In fact, the observed effects may delay the onset or the progression of chronic diseases involving the cardiovascular system. SPE, kaempferol, crocin from petals of *Crocus sativus* L., were shown to affect several targets, thus they may provide benefits in the early stages of chronic diseases such as atherosclerosis or hypertension.

In order to achieve the potential beneficial effects, the studied nutraceuticals need to be administered in the most appropriate way. For this purpose, SPE, crocin, and kaempferol may be provided, in future in vivo studies, through a proper pharmaceutical formulation.

In conclusion, we suggest that more investigation should be done to completely explore the potential cardiovascular protective activity of what until recently was considered waste material in the production of the “golden spice”.

## Figures and Tables

**Table 1:** List of primers for real-time PCR in H9c2 cells.

Gene	Primer
β-actin forward	AAGACCTCTATGCCAACAC
β-actin reverse	TGATCTTCATGGTGCTAGG
Nrf2 forward	CCATTTGTAGATGACCATGAG
Nrf2 reverse	GTATTAAGACACTGTAACTCGG
CAT forward	CAAGTTCCATTACAAGACTGAC
CAT reverse	TTAAATGGGAAGGTTTCTGC

**Table 2:** Cardiac activity of SPE, crocin, and kaempferol.

		Left Atrium			Right Atrium	
		Negative Inotropy			Negative Inotropy	Negative Chronotropy
Samples	Unit	Activity *^a^* M ± S.E.M.	EC_50_ *^b^*	95% conf int	Activity *^c^*M ± S.E.M.	Activity *^d^*M ± S.E.M.
**SPE**		36 ± 0.9				40 ± 1.4
	[µM]	[--]				[--]
	(*mg/mL*)	(*1*)				(*10*)
**Crocin**		73 ± 1.9			25 ± 1.2	20 ± 1.3
	[µM]	[5]	0.17	0.12–0.23	[100]	[100]
	(*mg/mL*)	(*0.0048*)	(*0.00017*)	(*0.00012*–*0.00022*)	(*0.097*)	(*0.097*)
**Kaempferol**		87 ± 2.1			29 ± 1.1	30 ± 0.9
	[µM]	[5]	0.15	0.083–0.63	[5]	[50]
	(*mg/mL*)	(*0.0014*)	(*0.000043*)	(*0.000024*–*0.00018*)	(*0.0014*)	(*0.0014*)

*^a,c^* Decrease in developed tension on isolated guinea pig left atrium (*a*) and on guinea-pig spontaneously beating isolated right atrium (*c*), expressed as percent changes from the control (*n* = 5–6). The left atria were driven at 1 Hz. *^b^* Calculated from log concentration–response curves (Probit analysis by Litchfield and Wilcoxon [24] with *n* = 6–7). When the maximum effect was <50%, the EC_50_ ino. values were not calculated. *^d^* Decrease in atrial rate on guinea pig spontaneously beating isolated right atrium at the concentration that produces the maximum intrinsic effect in parenthesis, expressed as percent changes from the control (*n* = 7–8). Pre-treatment heart rate ranged from 165 to 190 beats/min. Please note: the concentration that produces the maximum intrinsic effect (*a*, *c*, and *d*) and the potency (*b*) are expressed as µM for crocin and kaempferol and as mg/mL for all samples.

**Table 3:** Relaxant activity of tested samples on K^+^-depolarized guinea pig vascular (aortic strips) and non-vascular (ileum) smooth muscle.

		Vascular				Non-Vascular		
		Aorta				Ileum		
		K^+^ 40 mM	K^+^ 80 mM			K^+^ 80 mM		
Samples	Unit	Activity *^a^* M ± S.E.M.	Activity *^a^* M ± S.E.M.	EC_50_ *^b^*	95% conf int	Activity *^a^* M ± S.E.M.	EC_50_ *^b^*	95% conf lim
**SPE**			48 ± 1.3			89 ± 2.4	2.05	1.36–3.10
	[µM]		[*--*]			[*--*]		
	(*mg/mL*)		(*10*)			(*10*)		
**Crocin**			3 ± 0.1			15 ± 0.3		
	[µM]		[50]			[100]		
	(*mg/mL*)		(*0.0049*)			(*0.098*)		
**Kaempferol**		42 ± 1.6	69 ± 1.9			79 ± 2.4		
	[µM]	[50]	[50]	20.76	12.58–29.37	[5]	1.16	0.77–2.36
	(*mg/mL*)	(*0.014*)	(*0.014*)	(*0.0059*)	(*0.0036*–*0.0084*)	(*0.014*)	(*0.00033*)	(*0.00022*–*0.00068*)

*^a^* Percent inhibition of calcium-induced contraction on K^+^-depolarized (40 and 80 mM, respectively) guinea pig aortic strips and longitudinal smooth muscle at indicated concentration. The indicated concentration gave the maximum effect for the samples. *^b^* Calculated from concentration–response curves (Probit analysis by Litchfield and Wilcoxon [24] with *n* = 6–7). When the maximum effect was <50%, the IC_50_ values were not calculated. Please note: the concentration that produces the maximum intrinsic effect (*a*) and the potency (*b*) are expressed as µM for crocin and kaempferol and as mg/mL for all samples.
